# Evaluation of two social norms nudge interventions to promote healthier food choices in a Canadian grocery store

**DOI:** 10.1186/s12889-022-14370-8

**Published:** 2022-10-20

**Authors:** Selina Suleman, Molly Sweeney-Magee, Susan Pinkney, Kimberly Charbonneau, Kelly Banh, Ilona Hale, Shazhan Amed

**Affiliations:** 1 British Columbia Children’s Hospital Research Institute, 4480 Oak Street, Vancouver, BC V6H 3V4 Canada; 2 Department of Family Practice, 3rd Floor David Strangway Building, 5950 University Boulevard, Vancouver, BC V6T 1Z3 Canada; 3 Department of Pediatrics, University of British Columbia, 4480 Oak Street, Vancouver, BC K4-213 Canada

**Keywords:** Nudge interventions, Behaviour change, Healthy eating, Food choice

## Abstract

The objective of this study was to determine the impact of two nudge interventions on customers’ produce purchases at a rural Canadian grocery store. A pre- and post-intervention observational study design was used. Sales data were gathered before and after the staggered implementation of two nudge-based interventions to encourage produce purchases: grocery cart dividers to encourage shoppers to fill one-third of their cart with produce and grocery cart plaques with information about how many fruits and vegetables were typically purchased in the store. The proportion of total sales accounted for by produce was compared between baseline and implementation of the first intervention (Phase 1), between implementation of the first intervention and the addition of the second intervention (Phase 2), and between baseline and post-implementation of both interventions together. There was a 5% relative increase (0.5% absolute increase) in produce spending between baseline and post-implementation of both interventions (10.3% to 10.8%, *p* < 0.001, 95% CI 0.2%, 0.7%). Intervention phase-specific produce spending showed no significant change in the percentage of produce spending from baseline to Phase 1 of the intervention, and an 8% relative increase (0.8% absolute increase) in the percentage of produce spending from Phase 1 to Phase 2 of the intervention (10.3% to 11.1%, *p* < 0.001, 95% CI 0.5, 1.1%). Simple, low-cost nudge interventions were effective at increasing the proportion of total grocery spend on produce. This study also demonstrated that partnerships with local businesses can promote healthier food choices in rural communities in Canada.

## Introduction

Fruit and vegetable consumption in children is an important contributor to reaching and maintaining a healthy body weight. A diet rich in fruits and vegetables is also protective against many chronic diseases such as cardiovascular disease, type 2 diabetes, and cancer [1]. Canada’s Food Guide (CFG) encourages individuals of all ages to include plenty of fruits and vegetables in meals and snacks (four to eight servings a day for children and seven to ten servings a day for adults (age and sex dependent) [2]. Despite this recommendation, just 20.7% of the Canadian population ages one and older met or exceeded CFG recommendations for daily fruit and vegetable consumption [3]. Youth -specific research has reported adherence to these recommendations to be as low as one in ten Canadian children [4].

Healthy eating is an individual behaviour but there are many internal and external factors – e.g., biological, psychological, cultural, and social factors, as well as community and policy settings – that influence food choices [5]. Effective health promotion and childhood obesity prevention efforts must extend beyond a single sector and involve the whole-of-community so children and families see consistent messaging and are supported in making healthy choices where they live, learn, and play [6].

Sustainable Childhood Obesity Prevention through Community Engagement (SCOPE) is a community-based participatory research project in the province of British Columbia (BC), Canada. SCOPE developed Live 5–2-1–0, a multi-sectoral, multi-component childhood obesity prevention initiative centered on the evidence-based 5–2-1–0 message (i.e., five or more portions of vegetables and fruits, < two hours of recreational screen time, at least one hour of physical activity, and zero sugary drinks per day) [7, 8]. Using a collective impact approach, SCOPE partners with communities across BC to engage a range of community stakeholders (e.g., in local government, health, education, business) to share the Live 5–2-1–0 message and create healthier environments for children and families [7, 8].

Businesses are an integral part of a community and play an influential role in the health behaviour choices that are available, as well as the healthy messaging that is relayed to local residents. Grocery stores are one private sector setting where health promotion initiatives aimed at increasing fruit and vegetable intake can be mutually beneficial to both the patron (improving customers’ food choices) and the business (potential profitability of increased produce sales, a high margin product category) [9]. However, to date, grocery store interventions aimed at promoting healthy food choices through education and environmental changes have had mixed outcomes and have been mildly successful at best [10, 11]. For example, point-of-purchase interventions (e.g., interactive displays and brochures) alone were deemed ineffective in a systematic review of grocery store interventions, while studies that combined point-of-purchase interventions with changes to pricing, the availability of healthy food, promotion, and advertising showed stronger effect on promoting healthy food choices [10].

There is growing evidence of the effectiveness of interventions that utilize ‘nudges’ – a nudge aims to alter an individual’s behaviour in a predictable way without restricting one’s options when making decisions [9, 12–16]. Nudge interventions are based on dual-process models of behaviour which posit that behaviours result from the interaction of both an unconscious, automatic mode of processing (System I), and a conscious, slow, rational mode of processing (System II) [17]. Nudge interventions utilize heuristics to influence the automatic/System I mode of processing and decision-making. Grocery store nudge interventions may also leverage the social aspects of grocery shopping such as shoppers’ perceptions of what foods are common, normal or appropriate to purchase [18]. Examples of nudges in this setting include highlighting items using focused lighting, mounting shelf labels that advertise promoted items, and improving accessibility of products through product placement [9, 19, 20].

One nudge-based intervention that has demonstrated effectiveness in increasing fruit and vegetable purchases in supermarkets is the installation of partitions in grocery carts to designate a section for ‘fruits and vegetables’, thereby emphasizing social appropriateness of purchasing fresh produce [18]. Huitink et al. [18] showed that an inlay in grocery carts with messages about the vegetable purchases of other customers, and an allocated grocery cart partition for vegetables, resulted in a statistically significant increase in grams of vegetables purchased (900 g to 1120 g on intervention days). More recent work in Portugal exposed shoppers to a social norm message suggesting the healthiest families purchase 11 fruits and vegetables on each visit to the store. The researchers found that shoppers with the least healthy purchasing behaviours prior to the intervention were positively impacted by this intervention and increased the number of fruit and vegetables they bought.

Canada’s geography may play a role in access to healthy foods, especially for rural and remote communities [21, 22]. Nudge interventions may represent a mutually beneficial activity for grocery stores to support a community driven, collective approach to health promotion while increasing produce sales. However, grocery nudge interventions have not been explored extensively within the context of remote, locally owned grocery stores in Canada. This represents a significant gap in the literature as available evidence suggests rural residence may be a risk factor for having a poorer diet due to limited availability and higher prices for fresh produce [23, 24]. The objective of this study was to address this knowledge gap by using up-to-date data to determine the impact of two evidence-based nudge interventions on customers’ purchasing patterns and produce sales at a grocery store located within a rural Canadian community.

## Methods

### Setting & participants

The setting was a rural Live 5–2-1–0 partner community located in the Kootenay region of BC that has a population of approximately 7,400 (in 2016). The median age of the population is 48 years, the average household size is 2.2 persons and 64.4% of the population are married or in a common-law relationship [25]. The community has two grocery stores; an independent grocery and a store that is part of a national supermarket chain. Both stores were approached by a local member of the research team and invited to participate. The independent grocery store agreed while the chain store declined due to the challenges in accessing data required to measure outcomes. No human participants were directly involved in this study and no individual human data/clinical data was used.

### Intervention

**Fig. 1:**
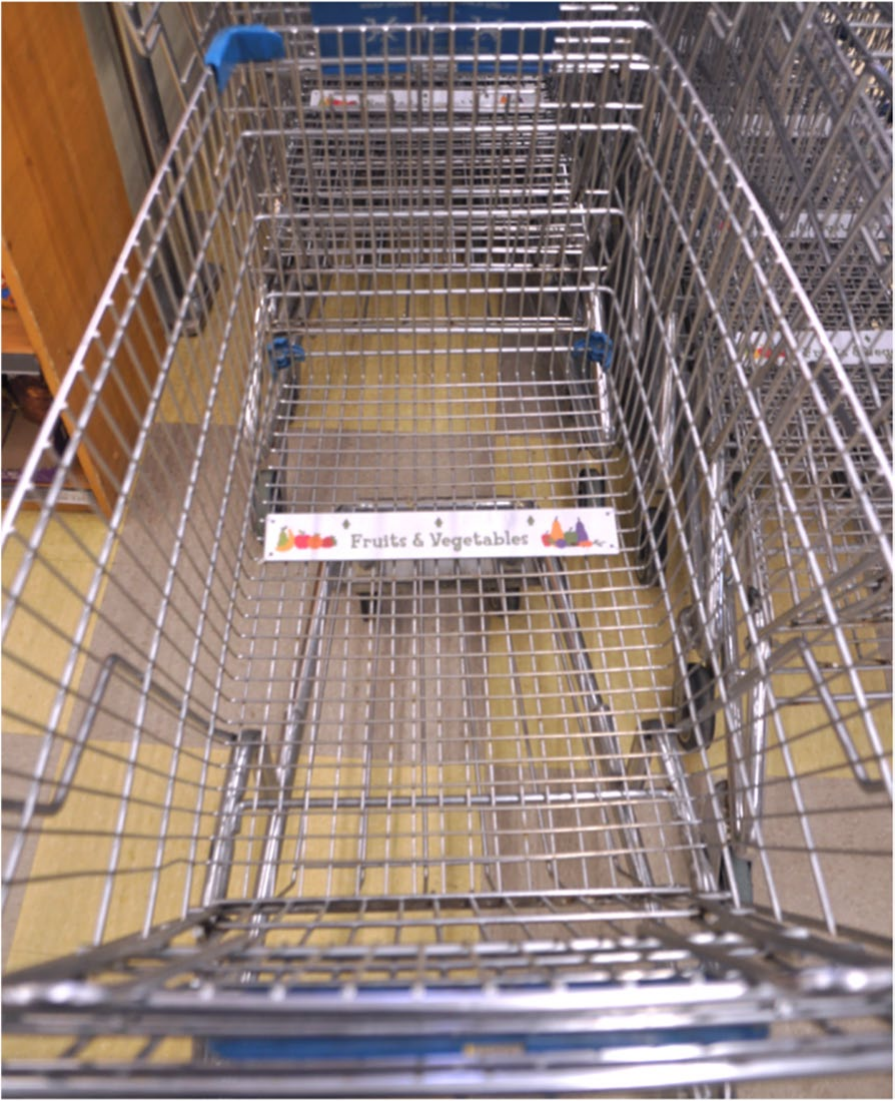
Grocery cart divider installed in Phase 1 of the intervention. Dividers remained installed in all grocery carts for the duration of the study

**Fig. 2:**
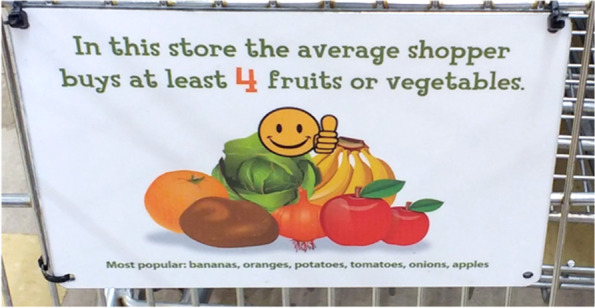
Informational plaque installed inside a grocery cart during Phase 2 of the intervention

The nudge interventions were based upon those developed by Payne and colleagues to subtly guide grocery shoppers to purchase more fruit and vegetables [26, 27]. The interventions were introduced in two phases:

1. In Phase 1 (February 2016 to January 2017) grocery cart dividers were installed to encourage shoppers to fill one third of their cart with produce. The dividers were made of a thin plastic strip that had text reading “Fruit and Vegetables” and colourful graphics, in addition to arrows pointing towards the front of the cart (Fig. 1). These flat strips were fastened to the bottom of the carts with zap straps at the junction of the middle and front thirds of the cart, creating a visual division in the cart. Dividers remained installed in all grocery carts for the duration of the study.
2. In Phase 2 (February 2017 to June 2018) plaques were installed inside all grocery carts with an informational message about how many fruits and vegetables were typically purchased in the store: “In this store the average shopper buys at least 4 fruits or vegetables” (Fig. 2). This information was based upon data collected in-store during the baseline time period (87 weeks).

### Study design & data collection

**Fig. 3:**
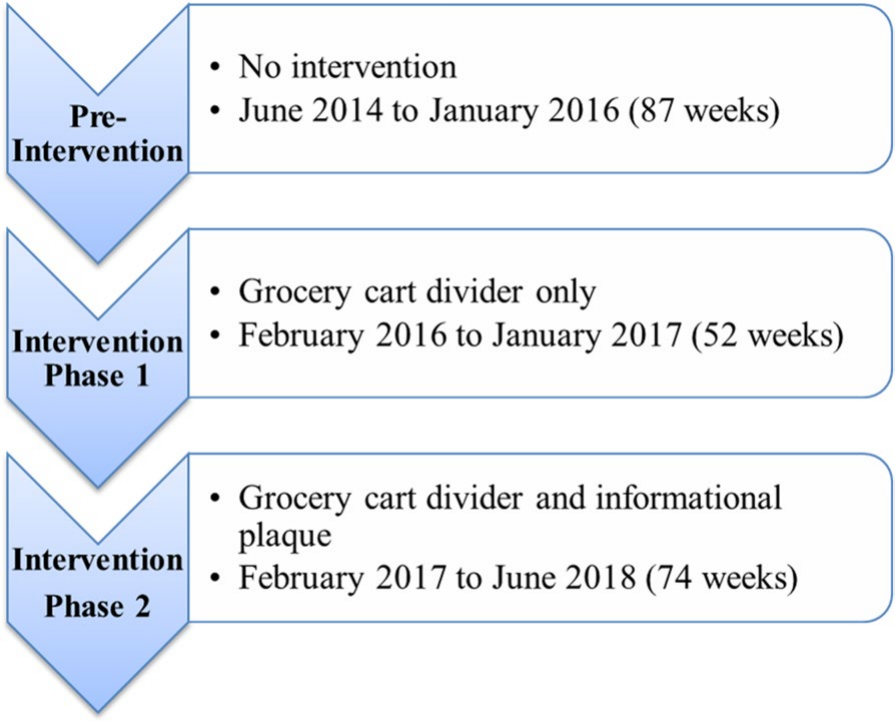
Description of study phases

A pre- and post-intervention observational study design was used. The primary outcome was the proportion of total sales that was on produce post-intervention relative to the same measure at baseline. The categories representing produce spending, grocery spending, and the proportion of total sales were obtained from grocery store sales reports for 213 weeks during the baseline and intervention periods. The baseline period accounted for the first 87 weeks of data collection (June 2014 to January 2016), and the intervention periods spanned the subsequent 126 weeks (Phase 1, marked by the installation of the part-cart intervention, spanned 52 weeks from February 2016 to January 2017, and Phase 2, marked by the installation of the cart plaques, spanned 74 weeks from February 2017 to June 2018) (Fig. 3). Weekly sales data were averaged and grouped into baseline and intervention periods (including two phases of intervention). The percentage of produce spending to total spending was analyzed to determine the change from baseline to the intervention period. The differing durations of all three phases was due to challenges in coordinating intervention implementation schedules with the store. Grocery sales data were also analyzed every three months to ensure sales were not adversely affected by the implementation of the interventions during the study period.

### Data analysis

Ethics approval for the study was obtained from the University of British Columbia Children’s and Women’s Health Centre of British Columbia Research Ethics Board (H15-01725). The proportion of produce sales compared to total sales was calculated each week at baseline from June 2014 to January 2016, and then each week during Phases 1 and 2, post-intervention. A two-sample t test was performed to assess whether there was a significant difference in the proportion of weekly produce to total sales at baseline compared to post-intervention. To assess whether there were any notable trends in seasonal produce purchasing, and to ensure that observed trends were consistent across baseline and intervention phases, monthly averages of the percentage of produce spending to total spending was calculated. Given the variable duration of the baseline and intervention periods, monthly averages were calculated and graphed for visual comparison across baseline and intervention.

A sensitivity analysis of matched months across all three time periods was also carried out to assess any effects of the variable length of each time period. The months of February to December in 2015 (baseline), 2016 (Phase 1), and 2017 (Phase 2) were compared using t tests.

## Results

### Produce sales pre and post intervention

**Fig. 4:**
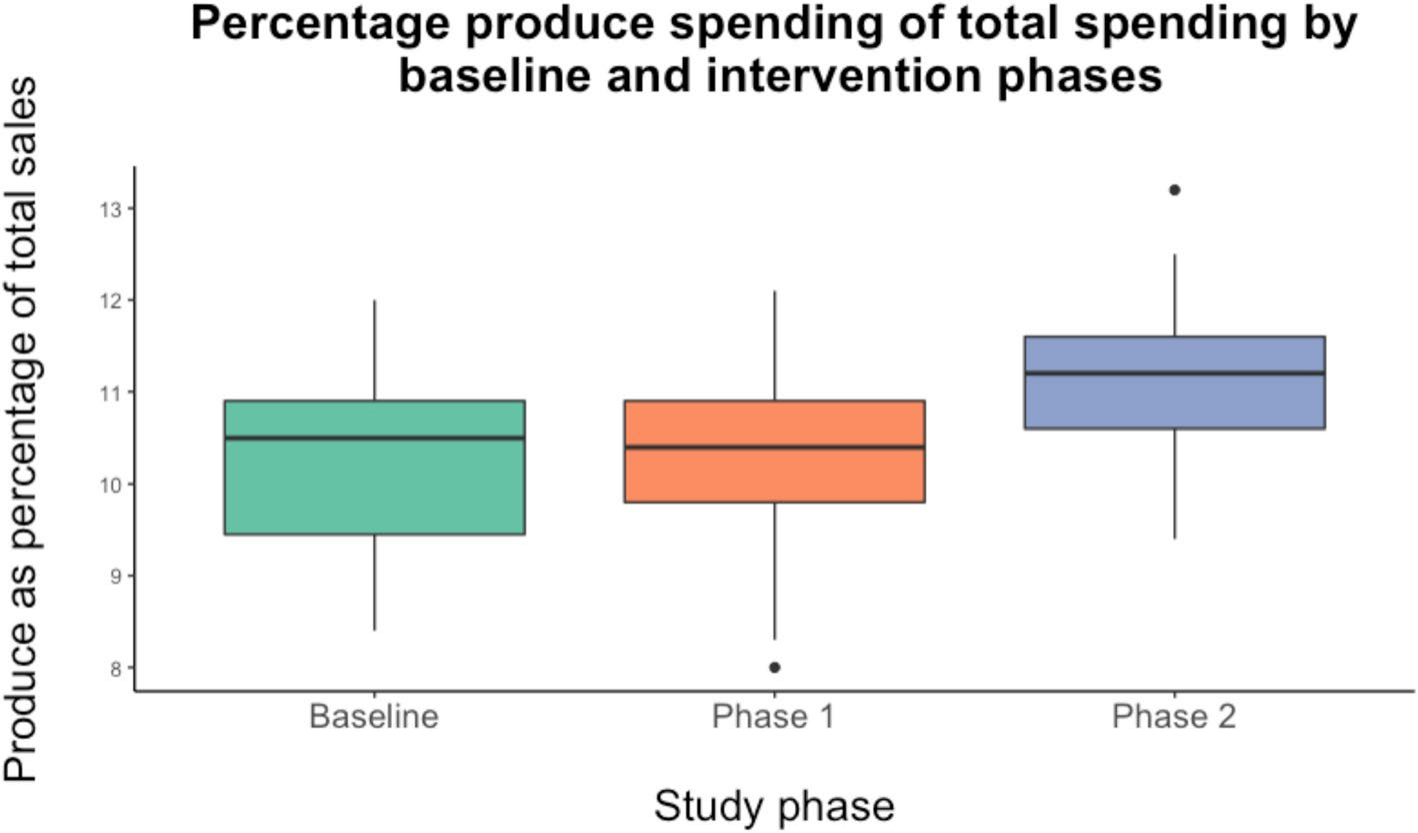
Percentage produce spending of total spending at baseline, and phase 1 and phase 2 of the intervention period (baseline to the intervention time period as a whole)

There was a 5% relative increase (0.5% absolute increase) in the percentage of produce spending to total spending when comparing baseline to the time period post-implementation of both interventions (10.3% to 10.8%, 95% CI 0.2, 0.7% absolute increase, *t*(211) = -3.48, *p* < 0.001, *d* = 0.49 (medium)). Intervention phase-specific produce spending showed no significant change in the percentage of produce spending from baseline to Phase 1 of the intervention (10.3% at both times, 95% CI -0.3 to 0.4% absolute increase, *t(*138) = 0.17. *p* = 0.86, *d* = -0.03 (negligible), and an 8% relative increase (0.8% absolute increase) in the percentage of produce spending from Phase 1 to Phase 2 of the intervention (10.3% to 11.1%, 95% CI 0.5, 1.1% absolute increase, *t*(124) = -5.45, *p* < 0.001, *d* = 0.98(large)) (Fig. 4).

### Produce sales as a proportion of total sales

**Fig. 5:**
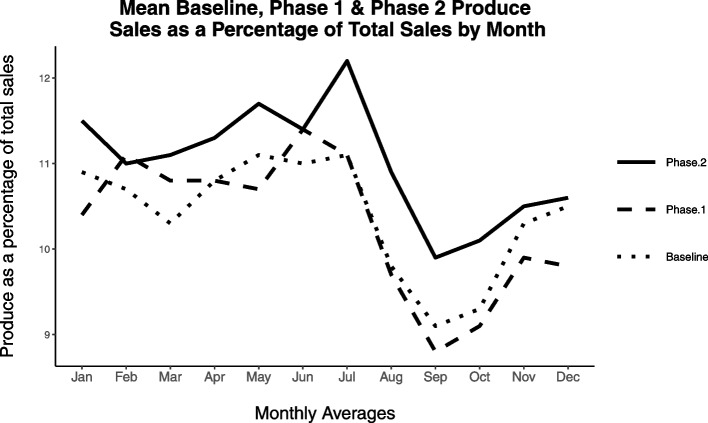
Mean baseline, Phase 1 and Phase 2 produce sales as a percentage of total sales by month (when two months of data from different years were available, these were averaged)

Average monthly percent of produce sales to total sales were calculated and graphed for each month in the baseline and intervention periods. Seasonal produce purchasing patterns were observed across the baseline and intervention periods, whereby produce spending increased over the spring and summer months and decreased in the autumn and winter months (Fig. 5). Thus, trends in produce spending displayed during the intervention period were consistent with seasonal produce purchasing trends during the baseline period. In addition, Phase 2 saw a sustained monthly increase in the proportion of total sales in the produce category with the introduction of the grocery nudge interventions. Overall, total sales did not differ between baseline and Phase 1 (*t*(138) = -1.06, *p* = 0.29, *d* = -0.19 (negligible) or between Phase 1 and Phase 2 (*t*(123) = 1.26, *p* = 0.21, *d* = 0.23 (small)).

### Sensitivity analysis

The sensitivity analysis using matched months from all three phases showed similar results to the main analysis. There was a significant increase in produce sales between Phase 1 and Phase 2 (10.3% to 10.9%, 95% CI 0.31 to 1.05, *t*(92) = -3.62, *p* < 0.001, *d* = 0.75 (medium)). However, there was no significant increase in produce sales from baseline when Phase 1 and Phase 2 data for the specified months were combined.

## Discussion

Our study sought to evaluate the effectiveness of nudge interventions in a rural Canadian grocery store to encourage consumers to purchase more fruits and vegetables. Our findings show that installing a grocery cart divider to create a designated space for produce in combination with a cart plaque with an informational message about typical fruit and vegetable purchasing practices in the store was associated with a statistically significant increase in the proportion of total sales that were for produce. Given the increase in produce sales with the addition of the plaque, it is possible that the impact on produce sales was primarily due to the plaque rather than the cart divider. Our results add to the existing evidence that nudges can be an effective strategy for increasing the purchase of fruits and vegetables in grocery stores.

Our results are similar to those of previously published studies that were used to form the basis of this study [27, 28]. While their outcome measures varied, these studies suggested that some combination of nudge interventions including grocery cart dividers and informational social norm messages pertaining to fruit and vegetable purchasing patterns had the potential to increase produce sales [18, 27]. Additionally, our finding of a potential benefit to implementing several grocery store nudge interventions at once is consistent with work by other researchers that suggests multiple concurrent nudges may be needed to influence purchasing behaviour [9, 29]. However, further research to examine the effect of the informational plaque alone would be needed to clarify its impact on produce sales relative to its impact when combined with the cart divider.

Installing the grocery cart divider on its own was associated with no significant increase in produce sales compared to baseline and there were intermittent periods during Phase 1 when the proportion of produce sales to total other sales dropped below baseline levels of this same measure (Fig. 5). These findings suggest that in a real-world setting, a grocery cart divider on its own may not be sufficient to nudge shoppers to buy more produce. However, the presence of both nudges in Phase 2 demonstrated a consistent and sustained increase in produce sales relative to total sales over the course of the study. This aligns with other research that found multi-layered interventions were effective at increasing produce sales [29]. As mentioned, future research could build on our work by examining the impact of the informational plaque alone to determine the precise effect of each intervention.

The present study has several strengths including a relatively low study cost, simple and feasible ‘real-world’ implementation, and secondary use of existing objective sales data that minimizes workload on grocery store owners. Additionally, each nudge was assessed at different time points but over a long time period that included all calendar months, allowing us to understand the impact of a single partitioned cart nudge versus a combination of the partitioned cart and the placard nudges. During the study time period, the Consumer Price Index (CPI) for fruit and vegetables and food in general was examined to identify any possible impact of changes in this on study results. Between June 2014 and June 2018, the CPI increased by 11.6 for fresh fruit and vegetables and by 10 for food in general [30]. The similarity in these increases make it unlikely that changing food prices impacted our results.

This study has several limitations. The pre- and post-intervention observational study design limits the ability to make causal inferences about the findings. It is possible that there were other events occurring simultaneously that led to the observed effects on produce sales. Furthermore, data were collected sequentially from baseline to intervention phases. Thus, there are no time-matched grocery store sales data for comparison, and it is possible that the observed results were a result of general changes in produce sales over time. However, as mentioned previously, the baseline and intervention phases spanned several seasons thus mitigating the likelihood of the observed effects being a result of seasonal changes in purchasing behaviours. Another limitation to this study is the use of total produce sales with no data on individual customer-level purchasing to determine the primary outcome measure. Use of aggregate sales data makes it unclear whether customers purchased a greater number of fruits and vegetables, or whether they purchased more expensive fruits and vegetables. Thus, future studies should investigate the effect of grocery cart dividers and social norm message nudges in a rural, Canadian context using customer-level produce sales data (such as grams of produce sales per customer, number of items of produce purchase and number of customers frequenting the store on average) and using a randomized control trial study design. Documentation of produce promotions during the study period would also help ensure any differences identified were due to the intervention(s). Lastly, our study did not collect data on produce consumption and therefore cannot ascertain whether an increase in produce purchasing translated into healthier eating at an individual level.

Grocery stores are one example of a private sector setting that can have a positive role in creating a healthier environment for local residents. Our study demonstrates that nudge interventions can be a feasible, relatively low-cost strategy to promote fruit and vegetable purchases, without negatively impacting total sales in a locally owned food business. Given the importance of fruit and vegetable consumption in promoting health and preventing chronic disease, the implementation of grocery store nudge interventions has the potential to positively influence the health of communities. Future research is needed to better understand whether an increase in produce purchases translates to an increase in the consumption of fruits and vegetables.

## Funding

The study described in this manuscript received no specific funding. There are no possible, perceived, or real financial conflicts of interest.

## Data Availability

The data that support the findings of this study are available from the participating grocery store but restrictions apply to the availability of these data, which were used under license for the current study, and so are not publicly available. However, data are available from the authors upon reasonable request and with permission of the participating grocery store. Please contact Molly Sweeney Magee, PhD, Live 5–2-1–0 Research & Evaluation Coordinator, BC Children’s Hospital (Molly.SweeneyMagee@bcchr.ca) with data access requests.
